# Hospital Meetings, &c.

**Published:** 1900-03-10

**Authors:** 


### HOSPITAL MEETINGS, &C.

### MIDDLESEX HOSPITAL.

Mr. Henry Custance presided at the quarterly general court of governors of the Middlesex Hospital on Thursday, 22nd ult. The annual report of the weekly board was read by the secretary-superintendent, Mr. F. Clare Melhado, and went exhaustively into the record of matters connected with the administration and management of the hospital. An unusually heavy strain had, the board stated, been put upon their resources by the temporary closing of similar institutions in the neighbourhood during the past year. The number of patients was not only in excess of those in the previous year when the hospital was closed for nine weeks, but was above the average. The in-patients totalled 3,611, the out-patients 49,817—together 53,428. The average weekly cost was £1 15s. 3½d. for in-patients, and 1s. 4¾d. for out-patients, as compared with £1 17s. 4d. and 1s. 5¾d. respectively in 1897—a substantial improvement. The "average stay" had increased both for medical and surgical cases. For the former it was 27·74, and for the latter, 31·2. The "index number," obtained by multiplying the cases by their "average stay," was 107,123, as compared with 100,093 in 1897, the last year with which comparison was possible. The ordinary income for the year was £19,493; the ordinary expenditure, £30,548. Legacies amounted to £6,768, in addition to £3,066 left to the cancer fund. There were also special donations to the cancer fund and the cancer investigation department amounting to £2,655. The extraordinary expenditure on building, new leases, and Samaritan fund and convalescent home amounted to £4,009. The balance-sheet showed an excess in expenditure over income of £8,296, as compared with a corresponding deficit in 1898 of £14,496. The working of the Samaritan fund and convalescent home resulted in a deficit of £2,715. The lady probationer fee fund and trained nurses' institute showed a considerably increased profit on the working, the balance transferred to capital being £1,106 in 1899, as against £848 in 1898. The nurses' earnings, £2,532, were slightly less; but the lady probationers' fees rose from £595 to £792. The rate of commission allowed to the nurses had been increased, and in future each nurse would receive in her first year's service 15 per cent., in her second 20 per cent., and in her third 25 per cent. of her gross earnings. Warm appreciation of the nursing staff was expressed by the board, and it was mentioned that retiring pensions had been awarded to Miss Taylor and Miss Newman, whose services as sisters extended over 21 years. A tablet had been placed in the chapel to the memory of Miss Frances Cox, who had been in the service of the hospital over 20 years, and was at the time of her death night superintendent. She had been succeeded by Miss Beaumont. Arrangements had been made for an extension of the leases of the Cleveland, Union, and Nassau Street properties for ninety-nine years from Midsummer, 1899, the rent remaining £1,183 as at present until 1907, when it would be raised to £1,400. During the year two of the vice presidents — the Duke of Beaufort and Viscount Gort—had died, and their places had been filled by the present Duke of Beaufort and by Sir Francis Grenfel, formerly a member of the board. The death of Mr. Noel, who had been a member of the board for nearly twenty years, was also mentioned with regret. The name of the Duke of Norfolk was put forward for election as a vice-president. Mr. Charles Davis had been elected to a seat on the board in succession to his father, the late Mr. Frederick Davis; Mr. Barrie Clayton and Mr. Horace Cockrell had been elected to fill other vacancies. Mr. George Howard had resigned the office of honorary auditor, which he had held for twelve years, and had been succeeded by Mr. Andrew Cunningham. Several important changes had taken place on the hospital staff and in the medical school. Sir Richard Powell had resigned the post of physician to the hospital and lecturer in the medical school, and his election as a consulting physician was recommended in recognition of his ervices for over twenty-one years. The appointment of Dr. Sidney Coupland, who retired in 1898 on becoming a Commissioner in Lunacy, was also recommended. The vacancy on the honorary staff had been filled by the election of Dr. Pasteur. Dr. J. Kingston Fowler and Dr. Pasteur had been appointed lecturers on medicine. Dr. Cecil Yates Biss's continued ill-health had necessitated his resignation of the posts of out-patient physician and lecturer on pharmacology and therapeutics after nineteen years' valuable service. In the latter of these appointments he had been succeeded by Dr. Wynter, and Dr Frank J. Wethered became fourth assistant physician to the hospital. Dr. H. Campbell Thompson had taken up Dr. Wynter's duties under the title of officer-in-charge of the electrical department. Mr. Alex. G. R Foulerton, F.R.C.S., bacteriologist to the hospital, had become lecturer on public health in the place of Dr. Pasteur, who resigned, and Dr. Stewart Crombie had been appointed to a lectureship on tropical medicine. Mr. A. M. Kellas, B.Sc., Ph.D., had become temporary lecturer in chemistry in the place of Dr. R. T. Plimpton, whose sudden death while in the active discharge of his duties was deplored. The newly created office of assistant anæsthetist had been conferred on Mr. Percy Noble, a former distinguished student of the hospital. Several of the junior officers, among whom were mentioned Mr. S. B. Hulke, surgical registrar, Mr. Reissmann, house surgeon, and Mr. Roberts, obstetric house physician, had been granted leave of absence for service in

South Africa; and Miss Morgan, the matron of the convalescent home, having been called out to join the Army Nursing Reserve, her duties were being temporarily fulfilled by Miss Zoe Brown, the assistant matron. The board reported with gratification that during the three years since the hospital and the medical school had been amalgamated the two bodies had worked together in perfect harmony, the arrangement proving an unqualified success. The number of entries of general students had steadily risen. The committee appointed to consider the necessity for alteration in the financial clauses of the scheme had reported that no revision—beyond a precautionary verbal amendment—was yet expedient. Mention was made of opening the new school buildings in March last. The laboratories, &c., had given the greatest satisfaction, while the establishment of a thoroughly equipped bacteriological department had provided for the systematic employment of such methods of examination in the investigation of disease. The board had long desired to further carry out the intentions of the founders of the cancer wards, and had come to the conclusion that the erection of the new cancer wing, with its ample accommodation for research laboratories, gave them an opportunity which should not be missed. Accordingly, a scheme for the special investigation of cancer was prepared, and a "Cancer Investigation Committee" had been constituted, whose functions included the supervision of research carried on in the laboratories, and the issuing of periodical reports on the work done; systematic bacteriological and histological examination would be made in every cancer case with a view to formulating a satisfactory classification of sarcomata, and to the investigation of the causes of carcinoma and sarcomata. Mr. Alex. Foulerton had been appointed the first director of the cancer research laboratories, with Mr. Hillier as his assistant, and Dr. Harold Clifford was medical officer and registrar. £20,000 was the sum estimated to be required for the adequate carrying out of the scheme. Towards this £1,605 had already been received; £465 was necessary for the fitting up of the laboratories and to provide the apparatus, and the yearly expenditure would be about £650. The committee had already entered on their duties, and had gratefully accepted Mr. Andrew Cringle's offer to undertake photomicrographic work, and any special section-cutting in connection with the department. Miss Bessie Davis had been appointed matron of the new cancer wing, into which the patients had been moved at the beginning of the year. The total cost so far had been £19,690. The convalescent home at Clacton continued to be of benefit to the work of the hospital. During the year, 816 patients—the largest number since its opening—had been received at the home, among these being fifteen of the nursing staff. The increase of 200 over the preceding year had been treated with practically the same ordinary expenditure—£3,711. It had been decided to arrange for the "open-air" treatment of specially selected male patients suffering from consumption, and five beds had provisionally been set aside for the purpose. The institution of the home as a permanent adjunct to the hospital having entailed upon the secretary-superintendent considerable extra work, the board had, in recognition of this and of his long service, raised his salary by £50.

Sir Arthur Watson moved the adoption of the report and its recommendations, going over and laying emphasis on many of the points mentioned.

Mr. Ormerod seconded, and in referring to the Cancer Investigation Department, said that if they could discover the origin of that fearful disease and the method of relieving it, they would have created a claim to the gratitude of the country and of the world. The report was adopted, and a vote of thanks to Mr. Custance for presiding terminated the proceedings.

### NATIONAL ORTHOPÆDIC HOSPITAL.

The annual meeting of the National Orthopædic Hospital, Great Portland Street, was held on Wednesday, February 28th, Lord Farquhar presiding. The accounts presented showed income £2,113, plus a legacy of £60, and expenditure £2,284. There was last year a small decrease in annual subscriptions—from £536 to £525—but the committee noted with gratification that this source of income had steadily increased since 1893, when it was only £227. A new rebuilding fund (No. 2) for the Bolsover Street improvements had been started, and Mr. W. H. Bishop, one of the trustees, had promised £500 a year to this fund till the plans were completed, and had already paid the first instalment. As well as the Duke of Marlborough, the president, Mr. Bishop, was absent in South Africa, and their safe return was earnestly hoped for. During the year 203 in-patients (122 children and 81 adults) had been treated. The out-patients numbered 1,384. Many of the in-patients were from the provinces, thus sustaining the national character of the institution. The report warmly eulogised the services of the Ladies' Committee and of the medical staff, and hearty votes of thanks to both were adopted by the meeting.

### ROYAL NATIONAL HOSPITAL FOR CONSUMPTION, VENTNOR.

The thirty-first annual meeting of the governors of and subscribers to the Royal National Hospital for Consumption and Diseases of the Chest (on the separate principle), Ventnor, was held on Wednesday afternoon, February 28th, at the offices, 34, Craven Street, Strand, the Attorney-General (Sir Richard Webster, Q.C.), chairman of the board of management, presiding. The annual report of the board submitted showed that the number of in-patients in 1899 was 880, an increase of 40 on 1898, whilst on December 31st last 229 medically accepted candidates were waiting their turns for admission. The eleventh block of buildings for 21 additional patients was opened for occupation on August 29th last by Princess Henry of Battenberg on behalf of the Queen, this increasing the number of beds to 153. The open-air treatment and its evolution had been carefully studied and developed by the administration. The total receipts were £21,547, which included legacies of £9,021, £584, and £500, whilst the ordinary expenditure amounted to £13,254, and the extraordinary expenses to £9,701, making the total expenditure £22,955. The latter includes the cost of the erection and furniture of the new block and electric lighting (of which a complete installation has been carried out) and other improvements and additions.

The Chairman, in moving the adoption of the report, regretted the absence of Lord Rosebery, their president, who had sent a letter announcing his absence from town. Referring to the losses sustained by the institution during the past year, Sir Richard feelingly alluded to the death of Mr. Neale Flower Horne, who had been deputy-chairman of the board for 28 years, and said it would have been quite impossible for him to have carried out his duties as chairman had it not been for Mr. Horne's valuable assistance and loyal service. They had also suffered very great loss by the death of Dr. Sinclair Coghill, who had been their senior physician for twenty-five years. He (the chairman) congratulated the subscribers upon the opening of the new block. As they would see by the report there were still an increasing number of patients waiting for admission, and as they hoped to develop still more the system of open-air treatment they had to look forward to finding an ordinary expenditure during the current year of about £13,000. In spite of the many and just calls now being made on the public for support, the board had good ground for appealing for further assistance in the work of the institution.

Lord Glenesk, in seconding the motion for the adoption of the report, said that he had found that although the calls on the public were so great the nation could still find money for such hospitals as this one. Consumption swept away annually by its ravages more people than did war, and in this war against consumption their institution at Ventnor was one of the bulwarks at the fore of the combat. In this war against consumption there never was peace, and the victim had been hitherto foredoomed, and was the cause of pain and suffering to others long before the result came which was formerly thought inevitable. They now, however, learnt that such results were preventable, and they were daily meeting with more success in the battle against the disease. He felt sure the public would come forward with its generous aid when once it was made clear how much the hospital wanted, and for what purpose.

The report was adopted, and Mr. P. B. Burgoyne was re-elected treasurer. On the motion of Lord Glenesk a cordial vote of thanks was accorded to Sir Richard Webster for presiding.

---

### GREAT NORTHERN CENTRAL HOSPITAL.

Mr. C. F. Cory-Wright, the deputy-chairman, presided at the annual general council of governors of the Great Northern Central Hospital on the 23rd ult. He expressed regret that they had not the pleasure of seeing their chairman (Sir John Dickson-Poynder), but he was sure they all admired his patriotism, loyalty, and devotion to Queen and country in leaving wife and family to take part in the unfortunate war we were engaged in in South Africa. He congratulated the meeting on the fact that they had now, for the first time, every ward open and every bed filled. That was an epoch of which they might well be proud. During the year the in-patients had numbered 1,923—an increase of 222 over 1898. The number of beds occupied was 130, and the average stay of each patient rather over 24 days. Their out-patients had risen in number to 31,189, as compared with 28,160 in 1898, an increase of 3,029, which showed the great progress they had been making. A certain proportion of their income—£1,742—was, so to speak, ear-marked; it was given with the express condition that it was to be devoted to the endowment fund. That being so, their total income for the year available for maintenance and management was £11,609, of which £3,651 was from legacies, while they had spent £12,517, £773 of which was "extraordinary expenditure," leaving a deficiency of over £900. Added to their former deficit of £4,999 they had therefore a total deficit or debt of £5,907. Of course, to set against that they had some very valuable assets on the other side. They had the land, the building, the furniture and fittings, and the appliances. And they had also £30,000 duly invested for endowment purposes. So altogether, though he did not like that debt of £6,000, still he thought they would compare very favourably with any other hospital in London. But more money was wanted. If they continued to spend £12,500 and only receive £11,600 they must either run into debt or close one of their wards—a backward step, and one he did not doubt they would agree would be a great calamity. During the year they had had to mourn the loss of their good friend Mr. Harkness. To fill the vacancies on the committee caused by this and the death of Mr. Murdoch in the previous year Mr. G. H. Alexander and Mr. J. L. Smithett had been elected. The report and accounts having been adopted, the nine retiring members of the committee of management were re elected, as were the hon. treasurer and hon. secretary. A special general council was then held, at which a special resolution was passed authorising the presentation of a petition to the Privy Council for a charter of incorporation for the hospital. A vote of thanks to the chairman terminated the proceedings.

### SEAMEN'S HOSPITAL SOCIETY.

Mr. Percival Nairne presided at the seventy-ninth annual court of the Seamen's Hospital Society, held at the Institute of Chartered Accountants on Friday, February 23rd ultimo. The report was read by the secretary, Mr. Michelli. The past year was a memorable one from the fact that the London School of Tropical Medicine had been established—a new feature in the development of the society. During the year more patients had been cared for than ever before, totalling all together 25,797. Of these 2,515 were in-patients, and 23,282 out-patients, and though mostly British subjects, they came from the ends of all the earth. A generous response had been accorded to the appeal for funds to extend the branch hospital and to establish the School of Tropical Medicine in connection with it. The income for the year amounted to £17,428, of which £12,783 was ordinary, and £4,645 legacies. The ordinary expenditure was £18,055, while £978 was spent on repairs, and £4,147 on the new laundry opened in May last. The receipts in aid of the new wing and the school were £16,274. Mention was made of the fact that the Secretary of State for India had contributed £1,000, in addition to the amounts already received from other Government departments; and that Sir Henry Burdett had announced that he would provide a travelling scholarship of £300 per annum for three years. A special committee had been appointed to devise a scheme on the lines of ordinary schools of medicine, and eventually the management had been handed over to a school committee. The following gentlemen had been appointed teachers: Drs. Patrick Manson, R. Tanner Hewlett, Andrew Duncan, Oswald Baker, L. Westenra Sambon, and Professor W. J. Simpson, Messrs. J. Cantlie Malcolm Morris, and E. Treacher Collins. The school was fully equipped and open for students on October 2nd. The new wing of the branch hospital was now rapidly approaching completion, and it was hoped would be open for patients early in the coming summer.

The Chairman, in proposing the adoption of the report, remarked that they expected in the course of a few months to have a public opening, with a prominent public man to perform the ceremony. So far the school had been very successful, and more students had come in than had been looked for, showing that there really existed a demand for such an institution. The matter which was causing the committee much anxiety was the condition of the "Dreadnought" hospital at Greenwich. In the old days, it would be remembered, their work was conducted in one of Her Majesty's ships. In 1870 they moved into the infirmary of Greenwich Hospital, which was placed at their disposal. They spent £10,000 on the drainage to put it into a proper state for their purpose, but now, after thirty years of active service, they found that the floors were in such a state of decay that they needed renewal. There were other repairs and alterations required, and the necessary expenditure was from £15,000 to £20,000. They had approached the Government with a view to getting assistance in the matter, but had received no encouragement to expect any help. It ought not to be forgotten that in the event of our being engaged in a war near our shores Greenwich Hospital was the place to which our sailors would be brought, and the committee felt that the Admiralty ought to keep it in a state to receive them. It had been considered whether they should erect an entirely new building, but there were strong sentimental objections to the removal from their present abode; and in the speaker's judgment the present was not a good time for erecting new hospitals, since he considered that modern sanitation was in a far worse state than that of thirty years ago. He could not believe that the system of ventilating their drains into the street, at almost the level of their neighbours' windows, regardless of the inconvenience or injury caused, showed progress but the reverse. The report was adopted, and Lord Hopetoun, Lord Strathcona, and Mr. Chamberlain having been re-elected vice-presidents of the institution, the proceedings terminated.

### QUEEN CHARLOTTE'S HOSPITAL.

The annual meeting of the governors and subscribers of Queen Charlotte's Hospital was held at the hospital on the 26th ult, at half past four p.m. The Earl of Hardwicke occupied the chair, and moved the adoption of the report in a few remarks commenting on its most salient points of interest.

The most important event in the year's annals was the opening of the new nurses' home by the Duchess of York, when, by her permission, two wards were named the "Mary Adelaide" and the "Princess May" respectively. The Committee of Management gladly availed themselves of the opportunity thus presented of acknowledging the deep obligation of the charity to the honorary services of Mr. Fred W. Hunt. This gentleman had not only designed and superintended the building of the nurses' home, but he had greatly improved and added to the old buildings of the hospital itself. There had been an increase of 38 in the number of in-patients, 1,150 being received. The rate of mortality was 4·3 per 1,000; of the five deaths which occurred one was due to septicæmia, and four to grave complications. The hospital had opened its doors without letters to the wives of the soldiers and sailors serving in South Africa, and about fifty had already received its benefits. The Princess Louise, President of the Soldiers and Sailors' Families' Association, had gratefully acknowledged this service. The expenditure of the institution had increased with the added accommodation, but it was proportionately less in comparison with that of previous years. Unfortunately the income, owing to the death of liberal subscribers, was £123 under that of the previous twelve months. The figures stood at £4,390 for outgoings, and £3,378 for ordinary revenue. The Prince of Wales's Fund made a grant of £300 to the hospital and a special grant of £50 to the convalescent home. The Metropolitan Hospital Sunday Fund awarded an increased grant of £312, and the Hospital Saturday Fund gave £91. The committee earnestly recommended the convalescent home to the generosity of subscribers and their friends. Mrs. Hardy's kind endowment of £200 permits the admission there of some poorer patient without the usual charge of 5s. a week. The committee had decided to federate the institution with the Royal National Pension Fund for Nurses with a view of encouraging permanent members of the nursing staff to provide an annuity for their old age. Dr. Grigg had been elected vice-president and consulting physician of the hospital upon his resigning his appointment as physician to the in-patients. The change thus made decided the committee to increase the number of the medical staff. Dr. W. Rivers Pollock and Dr. Wm. J. Gow had been appointed physicians to the in-patients; Dr. T. W. Eden, Dr. C. Hubert Roberts and Dr. Arthur F. Stabb physicians to the out-patients; Dr. W. P. Herringham physician (non-obstetric); Mr. Sydney Stephenson, ophthalmic surgeon; and Mr. Frank Morley, dental surgeon. The enlargement and improvement of the hospital was now approaching completion. The new wards, gained by adding another storey for the accommodation of the sisters, were now in occupation. The passenger lift, new steam disinfector and destructor, have proved most useful. Contributions to the building fund had reached £2,159, bringing up the total amount received for this purpose to £10,915. A further sum of £5,000 was needed to meet the originally estimated expenditure. Mr. Hunt, the honorary architect, was elected vice-president in recognition of his services, and the usual votes of thanks were passed unanimously. The proceedings, which were purely formal, then terminated.

### THE CANCER HOSPITAL.

Sir George Meason, the chairman, presided at the forty-ninth annual meeting of the Cancer Hospital (Free), Brompton Road, on the 28th ultimo. The accounts presented showed total ordinary income £8,833, and total ordinary expenditure £11,505. The legacies received amounted to £13,741, and the extraordinary expenditure on repairs, &c., to £508. The committee reported that, without sacrificing the comfort or well-being of the patients, or impairing the efficiency of the work, they had been able to effect an average saving of £5 per bed occupied as compared with the previous year. The in-patients numbered 827, the out-patients 1,637, whose attendances totalled 12,448. Mr. W R. Malcolm had accepted the office of treasurer rendered vacant by the death of Mr. H. L. Antrobus. At the suggestion of the Medical Committee, Mr. C. H. Leaf, F.R.C.S., and Mr. W. E. Miles, F.R.C.S., had been appointed assistant surgeons in order to cope with the increasing amount of work. The other changes in the staff were the appointment of Mr. R. G. Murray as assistant house surgeon, in place of Mr. C. H. Prichard, who had resigned on account of ill-health; Mr. Murray's promotion to the senior position on Mr. G. Q Richardson's resignation to take up duties at the Westminster Hospital; Mr. Vivian B. Orr's appointment to the junior post thus rendered vacant, and his resignation on being appointed to the Westminster Hospital.

In moving the adoption of the report the Chairman appealed for assistance in carrying out a scheme for the improvement in the nurses' quarters, an alteration which is much needed. The report was adopted, and in replying to a vote of thanks to the medical staff, Dr. Marsden mentioned that the Medical Committee had under discussion a plan which they hoped to complete in the near future for enlarging the pathological department, so as to give the pathologist, Mr. H. G. Plummer, further opportunities for extending his observations, which had proved most valuable.

### TOTTENHAM HOSPITAL.

The first annual general meeting of the subscribers and donors to the Tottenham Hospital was held on the 27th ult., Mr. C. F. Cory-Wright (the chairman) presiding. The report presented by the managing committee was the first since the new scheme sealed by the Charity Commissioners in November, 1898. At a meeting held in January, 1899, the election of twenty-one subscribers to form, with those nominated by the medical staff, the managing committee took place, and at the first meeting of that committee in February Mr. Cory-Wright was elected chairman, Mr. E. Patten Huggett vice-chairman, and Mr. J. P. Beavan treasurer. Twenty-four beds were opened for the reception of patients, but in June the committee decided to open a ward containing twelve cots for children. A garden party held in July resulted in £123 being contributed for the maintenance of this ward, in addition to £200 handed to the treasurer by the Ladies' Association for the general expenses of the hospital. The in-patients treated during the year numbered 530; the out-patients 3,766, or, counting dental cases and casualties, 9,157. The attendances of out-patients numbered 24,507; the daily average number of beds occupied was 29; the average cost per head per in-patient was £4 10s. 11d.; average cost per bed occupied, £82 16s. 0¾d.; average cost per in-patient per week, £1 11s. 10½d.; average cost of each out-patient, 1s. 6d. The income for the year, including £1,500 from the Prince of Wales's Fund, was £4,984—a result naturally regarded as gratifying and encouraging, especially as £1,122 was derived from annual subscriptions. The expenditure amounted to £3,141, of which £53 was for repairs and a fire-proof safe. The money collected in the neighbourhood for a Jubilee Commemoration Fund was to be devoted to the provision of a good laundry for the hospital, which would not only be a great convenience, but effect a material saving. Mr. W. D. Newton, for seven years secretary under the old scheme, had been appointed deputation secretary to the hospital. Regret was expressed at the loss to the institution of the services of Dr. Overend by removal from the neighbourhood, and of Dr. Watson by death. The latter's post as out-patient physician had been filled by Dr. Chappel. A new department for diseases of women had been created, and Dr. A. E. Giles appointed as honorary gynæcologist to out-patients. Miss Fleetwood had been succeeded as superintendent of nursing by Miss E. M. Fox.

In moving the adoption of the report and accounts the Chairman remarked that in the first year of an institution where drastic changes had been made there were always some difficulties to be overcome, but their first year under the new conditions had been a very satisfactory one. The fact that they had increased the number of beds occupied by 50 per cent. was a clear sign of progress. Though the average cost per bed was shown in the accounts as £82 16s., by the opening of the extra beds this had been reduced to £68 ; and when, as they hoped would very shortly be the case, another new ward was opened, the cost would be still further reduced. He was glad to know they had no debt ; he doubted if any other hospital in London could say the same. He objected to debt ; it was not honest, either in business or in charity. So, with the new wards they were opening, they must get more money from somewhere. And he felt sure that the more patients they had the more help they would get. The report was adopted, and the election of seven members of the committee proceeded with, resulting eventually in the declaration by the Chairman that Mr. Adair Roberts, Rev. Hay Morgan, Major Johnston, Mr. R. Colsell, Mr. H. Nield, Mr. E. Crowne, and Mr. Young were the successful candidates. A vote of thanks to the chairman closed the proceedings.

### ROYAL FREE HOSPITAL.

Lord Stamford, the vice-president, took the chair at the annual Court of Governors of the Royal Free Hospital, Gray's Inn Road, on the 28th ult. In moving the adoption of the report and financial statement he observed that that hospital had taken the lead in several valuable directions. It was the first to throw open its doors to lady students, a privilege which was now largely taken advantage of. He considered that the public owed them a great debt for that step, and said it might be that the time was coming when it would be possible to consider the question of properly-qualified women taking their places on the resident staff—a statement which elicited considerable applause from the large number of ladies present. Another very important departure, his Lordship went on to say, was in connection with their charitable work. It was there that the first lady almoner was appointed to go into the cases of those who applied for aid in the out-patient department. It was an advance upon the idea of an inquiry officer which was primarily of a negative character, with the idea of repelling the unworthy. This was work of a positive or active nature, and dealt with cases, not from the point of view of mere refusal, but considering how could the cause of real charity best be met. Their attitude was : "My poor friend, I'm afraid you've come to the wrong place, but let's see which is the right place for you," and the endeavour was made to find out how best assistance could be rendered, and to direct applicants to the proper quarter for help. In some cases a man was found on inquiry to be able to pay for the services of a general medical practitioner ; others were cases of utter destitution, fit only for the care of the Poor Law authorities ; while others still were recommended to apply to other sources of assistance—though in all cases first aid was given, in the interest of the medical profession, in order that peculiar or instructive symptoms might be noted In dealing with the financial statement he noted the sad fact that nearly half their income came from legacies—a very precarious source, since people declined to die in the precise years when their benefactions would be of most benefit. The average cost of each bed per week was only £1 7s. 11d., a result due to the very great care exercised by the committee. Their district was one of the very poorest in London, and had been shown to contain the very high proportion of 61 per cent. of the poorest class. The hospital was continually needing additions the thing just now urgently required was new nurses' quarters, those they had being very far from reaching the modern standard, and help to carry out this work was urgently needed.

Mr. Charles Burt, the chairman of the Weekly Board, seconded the motion, and briefly called attention to the work done during the past year. Their in-patients had numbered 2,117, and their out-patients and casualty cases nearly 35,000. Their total receipts were £11,930 (of which £5,853 was from legacies) and the expenditure £11,837—which, he thought, was running it quite as close as they ought. Although the accounts showed cash at bank £2,247, yet their outstanding liabilities to tradesmen for the current accounts actually reduced that to about £100 with which to begin the year. Still, they had been in that condition for some 72 years, and somehow the money had always been found. They strove always for efficiency—that first—and economy ; and among the 12 great hospitals in London with medical schools the Royal Free carried on its work at the lowest rates. This was not done by skimping; they paid their people properly. To keep going they collected what they could, and their friends, when they died, left them legacies, though he would point out that by giving them money during their lifetime they saved 10 per cent., and often 13 per cent., by being their own executors. In alluding to the fact that the Prince of Wales's Fund had given them £750 as an annual grant, and £500 as a special donation on the condition that this year they put the question of the nurses' quarters in the forefront, the speaker expressed the opinion that the Prince of Wales's Fund had done no harm to any hospital, but had done good to many, and a careful analysis of their accounts showed that not only had they not suffered since the Fund came into existence, but they had materially benefited by it. The fund had been of great service in the way not only of pointing out defects and helping to remedy them, but in the way of opening new wards and other improvements, as in their case.

The report was adopted, and Mr. W. W. Bramston Beach, M.P., having been appointed a vice-president, thanks were voted to the Committee of Management (which was re-elected) and to the staff and honorary officers for their services.

Mrs. Scharlieb, M.D., then moved a resolution which was unanimously adopted, expressing the thanks of the court to Mrs. Webb for the gift of a new museum and laboratories. These, which have been erected as a memorial of the late Miss Mabel Webb, formerly curator of the museum and assistant pathologist to the Royal Free Hospital, were then publicly opened by Lady Reay and inspected by those present. The former museum has now been converted into a laboratory capable of accommodating twenty students and fitted with modern appliances. The new floor which has been added to the building provides a museum fitted with shelves for 6,000 specimens, a laboratory for the use of the curator and the medical staff, and an assistant's room. Mrs. Webb has defrayed the cost of the structural alterations and additions amounting to £1,906. The expense of fitting up and furnishing—£425—is being defrayed out of a memorial fund which already amounts to £365, leaving a deficit of £60, towards which contributions are invited.

### ROYAL WESTMINSTER OPHTHALMIC.

Sir Charles Turner, chairman of the committee of management, presided at the annual general meeting of the Royal Westminster Ophthalmic Hospital on Friday, 2nd inst. The accounts presented showed an ordinary income for the past year of £2,411, and ordinary expenditure of £2,242. Legacies amounted to £2 101, and extraordinary expenditure—repairs, &c.—to £88. The committee in their report pointed out with satisfaction that annual subscriptions had increased from £762 in 1898 to £813 in 1899. Donations also showed an increase of £247, although during the latter part of the year there was an almost entire cessation of these, due, it was believed, to the demands upon public liberality by the various war funds. A point laid stress upon was the fact that the voluntary contributions of those who came to the hospital for assistance, found in the collecting boxes, amounted to £242—no less than one tenth of the expenditure on the maintenance of the hospital. The in-patients numbered 630, as compared with 585 in the previous year, the average stay being 14 82 days. The out-patients numbered 10,906, with attendances 28,427. These were the largest numbers on their records, and were in some measure due to the fact that children were sent by the School Board officers to have their sight tested. This enabled incipient mischief to be arrested and natural defects to be remedied or palliated. The increase accentuated the need for improved accommodation, and it was hoped that, despite the claims of the war, their building fund would not be overlooked. The Chairman briefly moved the adoption of the report, observing that they were in the happy position of having no history. The motion was agreed to, and the usual complimentary votes brought the meeting to a close.

**GREAT NORTHERN CENTRAL HOSPITAL,**

